# Explaining the reasons for not maintaining the health guidelines to prevent COVID-19 in high-risk jobs: a qualitative study in Iran

**DOI:** 10.1186/s12889-021-10889-4

**Published:** 2021-05-03

**Authors:** Neda SoleimanvandiAzar, Seyed Fahim Irandoost, Sina Ahmadi, Tareq Xosravi, Hadi Ranjbar, Morteza Mansourian, Javad Yoosefi Lebni

**Affiliations:** 1 Preventive Medicine and Public Health Research Center, Psychosocial Health Research Institute, Iran University of Medical Sciences, Tehran, Iran; 2 Department of Public Health, School of Health, Urmia University of Medical Sciences, Urmia, Iran; 3 Department of Social Welfare Management, Social Welfare Management Research Centre, University of Social Welfare and Rehabilitation Sciences, Tehran, Iran; 4 Islamic Azad University Sanandaj Branch, Sanandaj, Iran; 5 Mental Health Research Center, Psychosocial Health Research Institute, Iran University of Medical Science, Tehran, Iran; 6 Health Management and Economics Research Center, Iran University of Medical Sciences, Tehran, Iran; 7 Health Promotion Research Center, Iran University of Medical Sciences, Tehran, Iran

**Keywords:** COVID-19, Coronavirus, High-risk jobs, Prevention, Qualitative study, Iran

## Abstract

**Background:**

Although the workers in many occupations are at the greatest risk of catching and spreading COVID-19 due to assembling and contacting people, the owners of these occupations do not follow COVID-19 health instructions. The purpose of this study is to explain the reasons for not maintaining health guidelines to prevent COVID-19 in high-risk jobs in Iran.

**Methods:**

The present study was conducted with a qualitative approach among people with high-risk jobs in Tehran during March and April of 2020. Data were collected through semi-structured interviews with 31 people with high-risk occupations selected by purposeful sampling and snowballing. The data were analyzed using the conventional qualitative content analysis method and MAXQDA-18 software. Guba and Lincoln’s criteria were also used to evaluate the quality of the research results.

**Results:**

4 main categories and 13 sub-categories were obtained, including individual factors (personality traits, lack of self-efficacy, little knowledge of the disease and how to observe health norms related to it, misconceptions about health), structural factors (difficulty of access to health supplies, lack of supportive environment, weak laws and supervision, the poor performance of officials and national media), economic factors (economic costs of living, lack of government economic support), Socio-cultural factors (learning, cultural beliefs, social customs, and rituals).

**Conclusion:**

COVID-19 prevention requires intervention at different levels. At the individual level: increasing people’s awareness and understanding about how to prevent COVID-19 and strengthening self-efficacy in observing health norms, at the social level: highlighting positive patterns of observing health issues and training people about the consequences of social interactions during the outbreak of the virus, and at the macro level: strengthening regulatory rules and increasing people’s access to hygienic products and support for the vulnerable must be taken into account.

**Supplementary Information:**

The online version contains supplementary material available at 10.1186/s12889-021-10889-4.

## Introduction

The Coronavirus, which first spread in 2003, is a widespread family of viruses that lead to respiratory infections from a simple cold. COVID-19 is the newest member of this family [1, 2], which has rapidly spread around the world, and within a short period, it has become the most pressing health issue in the world. Therefore, the World Health Organization (WHO) declared it a pandemic [3, 4]. The spread of the disease was so rapid that it affected the economic, political, social, and even military aspects of all countries in the world [3, 5–9].

The number of patients and deaths with the disease is also increasing sharply [10, 11], and the mortality rate of this disease is reported to be 3.5% [2]. On April 19th, 2021, the total number of people infected with the COVID-19 in the world was 141 million, and the number of deaths was 3 million. The United States, India, and Brazil were among the countries with the highest rates of infection. The United States has the highest number of deaths from COVID-19 with 581,061 records. According to official statistics, Iran is one of the countries with the highest levels of COVID-19 infection with 2,237,000 infected cases and 66,732 deaths [12, 13].

The novelty of the COVID-19 virus and the lack of adequate knowledge on how to prevent and control it have made it challenging to prevent the disease. However, quarantine and maintaining health guidelines are some of the significant ways to prevent this disease [7, 8, 14, 15].

One of the locations that can be a place for rapid transmission of the virus is in the workplace [16]. In most of the working areas, people are prone to exposure to the new COVID-19 virus due to mass gatherings [17]. According to the WHO recommendations, restricting all social activities, including businesses, plays a key role in preventing the spread of the disease [18]. People are recommended to use face masks and disinfectants at work. They are also advised to avoid shaking hands with others and touching their eyes and nose, as well as to stay at home if they have an underlying disease or are suspected of having COVID-19 symptoms [19]. Some occupational groups that are directly in contact with clients and customers, such as health care workers [16, 20–22], transportation staff, restaurant staff, cemetery workers, commuting employees [20, 23], and hairdressers [24] are at greater risk, and they need to listen and act according to the health recommendations [16, 25, 26].

Despite having recommendations, some people and high-risk business owners do not follow the health advice related to COVID-19 [16, 17]. There may be several reasons for this. However, no independent study has investigated the reasons for not observing health guidelines on COVID-19. Liem et al., 2020, showed that migrant workers, due to the lack of reliable information in their language, did not understand the severity of the epidemic and how to protect themselves against it. Thus, they do not follow the health norms [27]. In the case of other infections and infectious diseases such as influenza, d’Alessandro et al., 2012, in a study on the reasons of groups exposed to influenza (H1N1) for refusing to vaccinate, showed that the existence of contradictory and ambiguous information on the effects of vaccines was a reason to avoid vaccination [28]. Adab et al., 2016, also presented in a study that awareness and attitudes about influenza (H1N1) can play a role in recommending health-based behaviors related to it [29].

Paying no attention to health instructions by high-risk business owners can jeopardize their health and the health of their families and even their customers. Therefore, it is necessary to explore the reasons for this phenomenon because by analyzing the reasons for the reluctance of following health tips, policymakers, managers, and health professionals can better plan to prevent this problem. Also, new studies related to the COVID-19 often employed quantitative, epidemiological, and clinical approaches [30–32], and there are few qualitative studies. Moreover, the reasons for not observing health issues related to the COVID-19 pandemic have been less researched. Therefore, the present study aimed to explain the reasons for not maintaining health advice to prevent COVID-19 in high-risk jobs in Iran using a qualitative method. The study was based on the experiences of people engaged in high-risk occupations in the period while quarantine rules and the lockdown was imposed in Iran. During this period, the activities in wedding halls, schools and universities, private educational centers, car sales centers, mosques, and other religious centers were banned. And at the same time, other occupations were allowed to operate by observing social and physical distancing and implementing health protocols.

## Method

### Design

This study was conducted with a conventional qualitative content analysis method [33, 34] in Tehran, the capital of Iran. No research has been conducted related to this issue in Iran so far, and thus, it has many unknown aspects that need to be studied. The qualitative approach is more appropriate to discover the hidden aspects of the problem; therefore, the researchers employed this approach. Since the purpose of the research is more to understand the phenomenon than to predict, and there is complexity in the subject of the study, the researchers used a qualitative approach and conventional content analysis method. Qualitative content analysis is one of the appropriate and coherent methods for analyzing textual data that is used with the aim of a better understanding of various phenomena [35]. In conventional content analysis, categories and subcategories are derived directly from interviews or group discussions and are not pre-formed.

### Participants

The participants in this study were engaged in high-risk jobs such as drivers, salespersons, hairdressers, bank employees, and municipality personnel, who did not follow the health recommendations related to COVID-19 prevention (Table 1). The interview guide was developed specifically for this study (Tale 2).

**Table 1:** Demographic information of participants

Variable	Dimension	Frequency (%)
Gender	Female	8 (26)
	Male	23 (74)
Age (Year)	18–38	8 (26)
	31–50	15 (48)
	Over 50	8 (26)
Marital status	Married	24 (77)
	Single	7 (23)
Job	Salesperson	10 (32)
	Bank employee	4 (13)
	Hairdresser	8 (26)
	Municipality employee	3 (10)
	Driver	6 (19)
Education	Under high school graduate	18 (58)
	High school graduate to BA	3 (10)
	Higher than BA	6 (19)

Due to the widespread and rapid outbreak of COVID-19, all employees in different occupations are at risk of contracting the disease, especially occupations that have direct contact with clients and customers. However, health care workers, laboratory staff, the staff of the tourism industry and transportation, hotel and restaurant staff, service center staff, service providers such as shopkeepers, due to being in direct contact with people or workplace contamination, are at greater risk. Preventive and control measures to protect these people against COVID-19 depend on the type of work and the risk of exposure, including the probability of contact with infected people and contamination of the workplace. Inclusion criteria included activity in high-risk occupations, non-compliance with COVID-19 health advice, and ability and willingness to participate in the study with maintaining safety norms.

### Data collection

Purposeful sampling was used to choose the participants. In a few cases (4 people), snowball sampling was used in such a way that the researcher asked people who did not follow the health recommendations and to introduce their other colleagues who did the same. To collect the data, semi-structured face-to-face interviews were used. Moreover, notes were taken during the interview whenever necessary.

After receiving the code of ethics from the Iran University of Medical Sciences, the researcher (the corresponding author of the article), the Data collection was started. In the first step, maintaining the principles of health advice, the researcher observed people in the community and identified those who met the inclusion criteria and interviewed them. The researcher then asked these people to introduce their other colleagues who did not follow and act according to the health norms in this pandemic and met the inclusion criteria. At first, the question was, “Do you use gloves, masks and wash your hands regularly?” And in the event of a negative response from participants, the interview process would begin. The researcher first introduced himself and gave a brief description of his resume to the participants. The objectives and necessity of the research and the interview process were then stated. Subsequently, when the participants gave their written consent, the main questions of the interviews were asked. Prior to the interview, all authors of the paper designed the general questions of the interview in one face-to-face session, two online sessions, and the question guide (Table 2). It should be noted that the order of the interview questions was different for each participant, and other research questions were asked according to the answer they provided. Participants determined the time and place of the interviews. The average interview time was 60 min, mostly done at workplaces and public places, such as parks. In all the interviews, the health principles related to COVID-19 were strictly maintained. The researcher used appropriate gloves and mask and provided these safety items to the participants, as well as kept the appropriate distance. All interviews were recorded with the consent of the participants. Data collection lasted for 40 days from March 10 to April 18, 2020.

**Table 2:** Checklist for questions of the interview guide

No.	Questions
1	What do you know about COVID-19? Please explain.
2	Please explain the symptoms, ways of transmission, and how dangerous COVID-19 is.
3	How do you think COVID-19 can be prevented?
4	Did you know that your job is one of the riskiest jobs in this COVID-19 pandemic? If yes, then why are you still working and have not stopped working?
5	Why do not you follow health guidelines?
6	Do you have enough access to hygienic products or are you reluctant to use them yourself? Please explain.
7	Does your employer warn you about observing the health issues? Do they provide you with hygiene items? Explain.
8	Do your colleagues observe health issues? Please explain.
9	What do you think about the performance of officials and television about the information and warnings they provide for people? Do they encourage you to consider health advice? Please explain.
10	What are the most important barriers for you to maintain the health norms? Please explain.

The data collection process continued until saturation was reached. Saturation in qualitative research is when data repeat and no new concepts can be derived from it [36]. In this study, the researchers reached saturation after the 25th interview, but several other interviews were done for accuracy, and finally, after 31 interviews, the collection of research data was completed.

In order to observe the ethical principles in research, written consent was taken from all participants and they were told that there was no constraint to participate in the research and that they could leave the interview whenever they wished. They were also briefed on the interview process and how the results would be published, as well as they were assured that their names would remain confidential in the publication of the results.

### Data analysis

The data analysis process was performed by the second and the corresponding authors using the Graneheim and Lundman’s method [33, 37]. They used the MAXQDA-2018 software for this purpose. In the first step, the researcher typed the texts of the interviews in Microsoft Word 2017 with the help of other research colleagues immediately after the first interview, on the interview day. In the second step, the text of the interviews was read carefully by the researchers three times to gain a general understanding. In the third step, all the texts of the interviews were read word by word with great care and patience, and thus, the initial codes were extracted. In the fourth step, the researchers put the codes, which were similar in meaning and concept, and could be placed in one group, into a subcategory and determined how they would relate to each other. In the fifth step, codes and subcategories were placed in the main categories, which were conceptually more comprehensive and abstract. Finally, in a joint session, the whole data analysis process was shared, and the opinions of all the authors of the article were used.

### Trustworthiness

To improve the quality of the results, Guba and Lincoln’s criteria were met [38, 39]. To enhance the credibility of the study, the researchers considered diversity in sampling and selected participants who had the greatest diversity in terms of demographic characteristics. To ensure the dependability of the research, the findings were provided to the participants with the telephone to express their views, which was eventually approved by all of them. Also, data analysis and findings were sent to 5 prominent researchers in the field of qualitative research, who also approved the stages of analysis and findings. To increase confirmability, while trying to avoid bias by researchers, all authors of the paper were involved in the process of analyzing and coding and all of them were present at the meetings and expressed their views. To increase transferability, a complete description of the entire research process was provided and participants were quoted directly. The research findings were also provided to seven individuals who had similar conditions as the study participants. But they were not included in our study. They also confirmed that they had similar experiences that the participants had.

## Results

A total of 31 individuals participated in this study. Their demographic characteristics are listed in Table 1. In the data analysis process, 345 initial codes, 13 subcategories, and 4 main categories were obtained (Table 3), which are described below.

**Table 3:** categories, subcategories, and codes extracted from data analysis

Categories	Subcategories	Codes
Individual factors	personality traits	Frustration, carelessness, impatience, laziness, risk-taking
	lack of self-efficacy	I can’t wash my hands after touching anything, I can’t wear gloves while driving, I can’t wear a mask because I feel short of breath, it’s hard for me to stay at home, I can’t work with my phone or do other things easily when I have gloves.
	little knowledge of the disease and how to observe health issues related to it	The use of one mask during the day, repeated use of a mask and disposable gloves, incorrect use of the mask, insufficient knowledge about how to wash hands properly, and lack of sufficient knowledge of symptoms, transmission ways and ways to prevent COVID-19
	misconceptions about health	Most old men take COVID-19 seriously, young people do not consider it as fatal, and people who had a previous illness die more. I am not sick. I am healthy. So nothing happens to me. I’ve heard that COVID-19 is like the common cold, not believing in quarantine
Structural factors	difficulty of access to health supplies	The high cost of hygienic products, lack of hygienic products, poor distribution of hygienic products in the city, crowded queue for distribution of hygienic products, poor quality of hygienic products
	lack of supportive environment	Not providing health facilities by the employer, lack of necessary hygienic products in the workplace, crowded work environment, non-observance of health norms by the customer or client, non-observance of health norms by colleagues
	weak laws and supervision	The lack of compulsion and pressure to observe health norms, the lack of rules to punish offenders, the lack of strict enforcement by officials, and the lack of adequate oversight for observing health norms
	poor performance of officials and national media	Not dealing with COVID-19 seriously, broadcasting contradictory messages, broadcasting different statistics, joking around about COVID-19 effects and consequences, not showing its serious consequences, and broadcasting TV shows (showing many people together)
Economic factors	economic costs of living	Reduction of income, being in debt, being a tenant, not having a job other than high-risk work, spending money on home and family
	lack of government economic support	Inadequacy of government assistance, fear of being fined by banks
Sociocultural factors	learning	Learning from officials as a model in media, Learning from neighbors as models, Learning from colleagues as models
	cultural beliefs	Fatalism, being labeled as fastidious and foppish by colleagues in case of observing health guidelines, being labeled as a coward by associates in case of observing health guidelines
	social customs and rituals	New Year (Norouz) parties, close relationship with each other, to have courtesies with colleagues

### Individual factors

This category consists of sub-categories of personality traits, lack of self-efficacy, little knowledge of the disease, and how to observe health advice related to it, as well as misconceptions about health.

#### Personality traits

The presence of certain personality traits, such as frustration, carelessness, impatience, laziness, and risk-taking in participants instigated them not paying attention to warnings and not acting according to the health norms. Observing all the health norms related to the prevention of COVID-19 required a lot of patience and perseverance, which was beyond the ability of some participants. Also, some of the other participants were inherently risk-takers, who due to their high risk-taking, did not see any necessity to follow health advice to prevent COVID-19. Moreover, the lack of hope for the future due to socio-economic problems caused some participants to be reluctant to survive. So they could not react appropriately to health issues.

*"I don't care at all if I get COVID-19, I don't care anything for a long time." (Participant 12)*

*"I don't have the patience to wear gloves or stay at home." (Participant 1)*

*"Sometimes I pay attention to it, sometimes I get lazy, I say take it easy." (Participant 5)*

*"I don't pay much attention to it, I'm not afraid of taking it, I'm not afraid of death, too." (Participant 8)*

*"I am disappointed with this world. Nothing matters to me. It doesn't matter if I die or survive, that's why I’m not hard on myself." (Participant 22)*

The presence of certain personality traits in participants made them reluctant to pursue health issues, which further endangered their health.

#### Lack of self-efficacy

Many participants stated that they were unable to observe the health principles associated with COVID-19, and this was due to the principles and rules that must be followed to prevent infection. The COVID-19 virus appeared all of a sudden and completely changed the lifestyle of people. To prevent it, many behaviors needed to be changed, such as washing hands, wearing masks, etc., which some participants find difficult, and they believed that they could not do these things.

*"As soon as I wear a mask, I feel short of breath." (Participant 29)*

*"It's hard for me to wash my hands several times in an hour." (Participant 16)*

*"I can't wear gloves when I’m driving." (Participant 8)*

*"If you want to be completely healthy, life will be very difficult and I can't do that." (Participant 11)*

There are many ways to transmit COVID-19, which can cause people to observe many health issues; this can lead to a decrease in self-efficacy in individuals because people do not see the potential to follow all the health tips.

#### Little knowledge of the disease and how to observe health issues related to it

This subcategory includes codes, such as the use of one mask during the day, repeated use of a mask and disposable gloves, incorrect use of the mask, insufficient knowledge of how to wash hands properly and lack of sufficient knowledge of symptoms, the way of transmission and prevention of COVID-19. This shows that people have little knowledge of COVID-19 and do not know how to observe corona-related hygiene requirements. Most participants had a low level of education and their jobs were such that they often spent many hours outside the home, which deprived them of adequate training to prevent COVID-19. As a result, many participants did not have a clear understanding of how to follow health tips, and in many cases, even if they did the desired behavior, they performed it incompletely, which could endanger their health.

*"I wear one mask every day and every time I want to eat something, I take it off and put it in somewhere. After having water or food, I use it again." (Participant 15)*

*"Before my customers come in, I tell them to wash their hands. So I get sure I won't take it even if they are sick." (Participant 23)*

*"When I see someone coughing, I walk away from them and I don't let them get close to me. So nothing threatens me anymore." (Participant 17)*

*"Those who have COVID-19 are sick very badly, so they can't get out for shopping, so I'm not too scared." (Participant 3)*

Despite the widespread publicity in the media, many people still do not have a clear understanding of COVID-19 and how to observe hygiene requirements, and this leads to the poor performance of preventive and hygienic principles and, as a result, the spread of the disease.

#### Misconceptions about health

Some participants had misconceptions about COVID-19, its characteristics, and how it infects and transmits, such misconceptions caused them to behave improperly and to neglect health issues. With the outbreak of the COVID-19 virus in Iran, a set of misconceptions was formed in society from the very beginning. Some people in the society considered themselves resistant to this virus, and this issue caused them to be hesitant to observe health norms properly. In some cases, infection of elderly people and people with an underlying disease led the young people and those without an underlying disease to mistakenly believe that they were resistant to the disease and did not need to be concerned about health guidelines. Others were skeptical of the COVID-19 death toll, believing it was an exaggeration and it was nothing more than a cold.

*"Most old men take COVID-19 seriously, while young people don't consider as fatal. Hence, I feel relaxed and carefree." (Participant 30)*

*"People who had a previous illness die more. I'm not sick. I'm healthy. So nothing happens to me." (Participant 22)*

*"I've heard that COVID-19 is like the common cold, we take it too much seriously." (Participant 19)*

*"If I wash my hands regularly and don't eat anything outside, I won't take COVID-19." (Participant 13)*

The misconceptions about COVID-19 have led many participants to believe that they are protected from the disease, which makes them less likely to observe health advice.

### Structural factors

Structural factors refer to the issues that are beyond the control and depend on the will of the individuals, and even if the individual is willing to address health issues, these factors can prevent it. This category includes the subcategories of difficulty of access to health supplies, lack of supportive environment, weak laws, and supervision, the poor performance of officials, and national media.

#### The difficulty of access to health supplies

Many participants stated that they were more inclined to observe health advice if there were health supplies. But in the community, scarcity, unfair distribution, and poor quality of health products, and crowded shopping malls were barriers to do so. The distribution of proper hygiene products to prevent the spread of COVID-19 has not been done properly and the government has not used an appropriate way for the public to have access to it. Because, on the one hand, the cost of hygiene products is too high for many people. On the other hand, hygiene products have not been distributed in the right amount in the city. So to get it, you have to search for hours in the city, and if you find, you have to stand in line for hours, which can have a great impact on not using hygiene products.

*"I like to observe, but if I want to be very hygienic, I have to spend all the money I make to buy hygienic products." (Participant 2)*

*"At first, I was very sensitive and looked for masks and gloves, but no pharmacies had these items so I let it go." (Participant 4)*

*"In the whole city, only a few places are arranged to distribute hygienic products. If you want to go, you have to wait in line for two hours. With these working conditions, I can't stand in line, although the queue itself is very dangerous because everyone is close to each other and not far enough from each other."(Participant 17)*

*"Two or three days ago, I took a pack of gloves, and as soon as I wore them, they were torn."(Participant 13)*

Therefore, one of the main reasons for not observing the health norms among the participants was the difficulty of access to health supplies. Health officials in Iran have not been able to create a suitable mechanism to provide easy access to hygienic products for all people.

#### Lack of supportive environment

Most participants stated that in the workplace, there is no support for following the health norms as well as there are many barriers that prevent them from observing health instructions. These barriers include having inadequate health supplies and necessary hygienic products in the workplace, overcrowding, not observing health issues by the clients and customers, and not observing health issues by colleagues.

*"I'm a taxi driver. Taxi drivers’ organization should have given us masks and gloves, but they didn't give us anything." (Participant 6)*

*"I’m a bank employee. From the beginning, they agreed to give us a hand disinfectant solution, but so far they haven't done anything. Maybe I would have followed health protocols if they had given us the equipment." (Participant 9)*

*"Our workplace is always crowded. If we observe health norms ourselves, it is useless when others do not observe it. We warn so many times a day, but no one listens. So I let it go." (Participant 9)*

*"I was very sensitive and observant, but when I saw that none of my colleagues observed health issues, I got disappointed." (Participant 21)*

Therefore, the lack of a strong supportive environment in the workplace and the lack of health supplies in it as well as the reluctance of the colleagues not observing health norms by were the main reasons behind it.

#### Poor laws and supervision

During the COVID-19 pandemic, the Iranian government, like other countries, failed to enact strong laws to make people comply with health rules. Therefore, many people did not feel any obligation to comply with the protocols announced by the Ministry of Health, and in many cases violated these protocols because they did not face any fines. Many participants criticized the laws and rules related to health issues and the way of supervising health issues, stated items, such as the lack of pressure to observe health issues, the lack of rules to punish offenders, the lack of strict enforcement by officials, and the lack of adequate oversight for observing health advice.

*"We Iranians are accustomed to being forced to do something, otherwise we don’t observe anything. Maybe if they forced us to observe rules, I would do it." (Participant 23)*

*"Everyone behaves the way they want. They did not make any laws to penalize those who do not observe health issues. They only fined those who had moved from their city to another city." (Participant 31)*

*"In the media, officials tell us to observe health advice all the time, but they never monitor. If they have more control, everyone will have to observe." (Participant 28)*

*"If they had set the law and had supervised it, both I and all my colleagues would have observed." (Participant 7)*

Therefore, the participants mentioned the lack of strong laws and poor supervision as one of the reasons for not observing health rules.

#### The poor performance of officials and national media

Many participants stated that they were dissatisfied with the performance of the authorities and the national media in reflecting issues related to COVID-19. Because they played a role in not observing health norms by not dealing with COVID-19 seriously, broadcasting contradictory messages, broadcasting different statistics, making fun of COVID-19, not showing its serious consequences, and broadcasting TV shows (showing many people together).

*"Many officials didn't take COVID-19 seriously at first, and we don't take it seriously when we see they don't take it seriously." (Participant 11)*

*"We have heard so many strange statistics that we no longer believe in anything. That is why I say that whatever happens is not important." (Participant 12)*

*"On TV, they telecast different opinions every day, one day they say to wash your hands a lot, another day they say wash a little. One day they say when you go home from work, wash your clothes, another day they say don't wash. One day they say not everyone should wear a mask, another day they say everyone should wear a mask. We are human beings, we got tired."(Participant 25)*

*"At first I took it very seriously and observed it, but when I saw the officials all joking around about COVID-19, I somehow concluded that this is not such a dangerous disease." (Participant 18)*

*"On television, they say don't gather in crowded places, but they put a hundred people in a small place in their programs. Well, when a person sees these, he feels that he doesn't need to be so observant." (Participant 10)*

Therefore, the officials and the national media, who could have played a significant role in informing the people, with their poor and conflicting performance, not only discouraged the people to maintain health norms, but also discouraged them from doing so.

### Economic factors

This category consists of the subcategories of economic costs of living and lack of government economic support. Iranian society has been under economic pressure in recent years due to sanctions, and this has led to the prioritization of the economy and lack of public attention to health issues.

#### The economic costs of living

In recent years, Iran has faced a difficult economic situation due to US sanctions. Thus, providing necessities for many people has become a major problem. This and the aftermath of COVID-19 left many businesses half-closed and people with lower incomes are already struggling to meet their needs. Most of the participants, especially those who were in freelance jobs, stated they could not close their high-risk jobs due to the economic pressure on their families and inevitably they had to go to work.

*"My income has been declining since the outbreak of the coronavirus. I stayed home for a week, but I saw that this was putting a lot of pressure on my family. So I went back to work." (Participant 27)*

*"I am a tenant. If I can’t pay the rent at the end of the month, my landlord will complain a lot. I don't have any other income, so I have to do vending." (Participant 16)*

*"I'm paying for a family of six. If I don't go to work, how can I earn and pay for their expenses? I know it's a bad situation and I may get sick and my family's condition may get worse, but I have no choice."(Participant 15)*

*"Anyone who comes out in this condition and puts his life in danger, be sure, it’s because of the financial pressure, otherwise no one will like to get COVID-19." (Participant 4)*

Thus, the high cost of living and the lack of income in these critical circumstances forced many participants to go to work despite being aware of the dangers that might threaten their health.

#### Lack of government’s economic support

The Iranian government failed to support the lower classes economically as needed during the COVID-19 crisis. So many participants were forced to work despite realizing the risky conditions that could threaten their health in order to be able to cover living expenses. With COVID-19, many businesses fell and many families were under a lot of pressure. Under these circumstances, most of the participants expected the government to provide them with more financial assistance, but they said that the government’s assistance was very small and they were even pressured by banks to pay their loan installments. So they had to work in their high-risk jobs.

*"The government didn't help me as a vendor, they tell us to stay home all the time, they don't know about my life." (Participant 7)*

*"Living costs have risen so much that with the little help the government is giving, we can't make a living, so people like me are forced to go to work in these bad conditions." (Participant 21)*

*"I owe to the bank and I have a monthly installment. If I don't pay my installment, I have to pay a fine. Instead of thinking about me, the government is just telling me to observe health issues. I come to work due to fear of being fined so that I can pay my installments at the end of the month."(Participant 24)*

The inadequacy of the government’s financial assistance and not considering the conditions of the families have forced many people to continue their work due to financial difficulties.

### Socio-cultural factors

Social and cultural factors always play a significant role in not observing health issues, and without knowing them, it is not possible to provide a complete explanation for the reasons for not observing health norms. The category of social and cultural factors in this study includes subcategories of learning, cultural beliefs, and social customs and rituals.

#### Learning

At the beginning of the outbreak of COVID-19 in Iran, many people in the society, even the country’s officials, did not heed the warnings of health experts, and their inattention to health issues and non-compliance with health rules in society had become a model. As a result, many people learn to disregard health issues by looking at society. Some participants told that when they saw officials, neighbors, colleagues, and others with whom they associate did not maintain health guidelines, they somehow followed them as a model and learned from them not to observe health norms.

*"I see a lot of officials on TV who have ten reporters in front of them but they don't use a mask. I say to myself it is not necessary; I have such a job that no more than ten people gather in my shop." (Participant 12)*

*"Most of my colleagues don't observe health norms. Well, when I see them, I get kind of relieved and I don't observe, too." (Participant 16)*

*"When I see our neighbors who don't observe health tips, I tell myself that I’m like them, whenever they observe, I do too." (Participant 5)*

Since many people in Iran do not observe the health tips related to COVID-19, this non-observance of health issues has become a model; So that people learn from each other not to follow the health tips.

#### Cultural beliefs

In Iran, certain cultural beliefs and behaviors cause people to pay less attention to health issues. Fatalism, unfaithfulness to the fact that problems can be solved by observing health issues, and being labeled as fastidious and foppish by colleagues if health issues are observed, are part of these beliefs and behaviors. Some of these beliefs are rooted in religion because most followers of Islam believe that life and death are in the hands of God and our behavior as human beings does not affect the time of our death. So they did not see the need to observe health norms. Also, some other beliefs are rooted in the culture and socialization of people because, in Iranian society, non-observance of health issues is considered as courage and its observance is considered as a kind of fear, and those who observe health issues are labeled as cowards.

*"Death and life are in the hands of God. If God wills, I will get sick even if I don't get out of my room; but if God doesn't will, if I go to the hospital and shake hands with COVID-19 patients, I won't get sick, so we shouldn't take it too hard." (Participant 8)*

*"I put on a mask for a few days, but my friends were making fun of me and saying, 'When did you get so fastidious?', so I took it off." (Participant 15)*

*"One of our colleagues was wearing a mask and gloves and was not talking to anyone. The rest of the colleagues were making fun of him, saying he was coward and afraid of death." (Participant 27)*

*"From day one, God has decided how we die, so whatever is supposed to happen will happen, whether I observe or not." (Participant 22)*

The belief that the destiny of people’s lives has been predetermined was prevalent among the participants. Also, in Iranian society, those who observe health issues are usually stigmatized as being fastidious and coward by those around them, and this can play a role in not observing health rules.

#### Social customs and rituals

COVID-19 prevalence in Iran coincided with New Year, which has its own special social and cultural conditions. Most of the participants said that due to social conditions and customs such as New Year parties, close relationships with each other, and having courtesies with colleagues it was difficult for them to maintain health advice.

"I, as a vendor, have been waiting all year for Norouz (Iranian New Year) to earn money for costs of several months. Now I can't stay home and let it go, although my sales have decreased a lot." (Participant 20)

"Everyone expects you to visit them on Norouz (Iranian New Year) vacation. I can't tell my mother that COVID-19 has come and we can't visit you." (Participant 6)

"We Iranians have a lot of courtesy. It happens to me many times that my colleague used my tools, but I couldn't tell him anything." (Participant 28)

"Everyone loves to congratulate each other on the New Year. When my colleague comes to kiss me, how can I tell him that he shouldn’t kiss me, you might get sick? I’m sure he gets upset. “(Participant 30)

There are strong kinship patterns in Iran and it plays a strong role in the communication and behavior of people. Therefore, most people gather at special parties and ceremonies and their presence are mandatory and if someone is absent they will be blamed by relatives. In the New Year, despite having many warnings from health experts, people could not refrain from attending family ceremonies due to the pressures of social customs and this made many problems for observing the health rules. The existence of specific social customs as well as the coincidence of the COVID-19 prevalence with New Year in Iran made it more difficult for Iranians to observe health guidelines.

## Discussion

This study was conducted to explain the reasons for not observing health tips to prevent COVID-19 in high-risk jobs in Iran using a qualitative method. According to the research results, various individual, socio-cultural, economic, and structural factors were influential on the non-observance of health advice and a low number of preventive behaviors among people with high-risk jobs.

Personality traits, including carelessness, impatience, laziness, risk-taking, and frustration were among the individual factors that led to the negligence of warnings and non-observance of health issues. Several studies have shown that personality traits can be effective in neglecting health behaviors. In the study of Lotfi et al., 2013, being unmotivated was mentioned as one of the factors influencing the non-use of condoms among prostitutes [40]. Lack of self-efficacy was another contributing individual factor for not observing the health rules. Hence, many people find themselves unable to observe health those health norms.

In the present study, little knowledge of the disease and health guidelines is one of the main subcategories and results of the study in individual issues. In line with this finding, Liem et al., 2020, stated that one of the reasons for not following the health norms and not protecting oneself against COVID-19 was the lack of correct information and not understanding the danger of the disease [27]. According to the study, despite global concerns and media attention to the issue of COVID-19, many people still do not have a clear understanding of COVID-19 and its consequences. Therefore, it leads to inaccurate prevention strategies and health rules. This finding is consistent with the results of the study of Taghrir (B) et al., 2020, in which less than half of the respondents stated that they had received all the information about the COVID-19 [41]. However, the population in their study was medical students who have higher health literacy than the samples studied in the present study. Regarding the knowledge and understanding of the disease in a study in Canada, the results showed that the knowledge and understanding of the risk of the disease are effective in observing the preventive behaviors for COVID-19 [42]. In the studies of Smith et al., 2020, in the United Kingdom and Carlucci et al., 2020, in Italy, low perception of risk played a role in non-adherence to home quarantine and health norms [43, 44]. Besides, in the case of influenza, one of the reasons for people not being vaccinated was the ambiguity due to access to multiple and contradictory sources of information about the consequences of vaccination [28]. All the discussions under the category of little knowledge of the disease and health guidelines related to it can be raised in the context of health literacy. The important aspect is the ability to use health knowledge and information provided to the public by the responsible organizations. At present, information related to COVID-19 is available to the public, but it is not adequate. Because, people must make well-informed decisions, and this decision-making requires having the skills and abilities to use the information properly. If the level of public health literacy leads to the ability to use the information correctly, the level of compliance with preventive behaviors for COVID-19 will be higher. Misconceptions about COVID-19 were another interesting result of the research. The ambiguity of the cause and the prevalence of COVID-19, on the one hand, and the inconsistent and constantly changing information due to media domination, on the other hand, had led to misconceptions about COVID-19. It led many of the participants to see themselves resistant to the disease, which made them less likely to observe health norms. In the study of Vázquez et al., 2020, the results indicated that the misconception that COVID-19 was not associated with asthma played a major role in non-compliance with health guidelines [45]. A Ugandan study on misconceptions and the risk of COVID-19 found that participants believed that men were more susceptible to COVID-19 than women and children [46]. In a study by Mekonnen et al., 2020, in Ethiopia on factors related to false beliefs of society about COVID-19, the results showed that job status, especially unemployment and lack of knowledge about the number of COVID-19 patients, was associated with misconceptions about the disease [47].

Structural factors were another result of the study. One of the main reasons for not observing health norms in participants was the difficulty of access to medical supplies. Accessibility means the opportunity and the ability to use services. Numerous studies have examined the impact of accessibility on public health [48, 49] because access to health services is one of the main goals of policymakers and a good way to achieve justice in the health sector. The results of the study showed that access to health services and supplies needed to deal with COVID-19 was undesirable, and people considered non-observance of health rules as a response to the feeling of injustice in the distribution of these services and supplies, indicating that the authorities of healthcare in Iran have not been able to create a suitable mechanism to give people access to these health supplies. In this regard, Emanuel et al., 2020, showed that governments and policymakers should put in all their effort to prevent the shortage of medical resources during the COVID-19 Crisis [50]. A study by Nzaji et al., 2020, in the Democratic Republic of the Congo demonstrated that dissatisfaction with the actions of the Ministry of Health in providing services and the provision of necessary medical equipment was effective in non-compliance with health orders [51]. Also, participants mentioned the lack of strong laws and poor oversight as another factor in the non-observance of health guidelines. The COVID-19 experience in Iran showed that health protocols notified by health institutions and the government did not have strong enforcement and oversight and were often not maintained. Furthermore, irresponsible people who did not follow the health norms did not get penalized. At the same time, the implementation of quarantine and traffic control as a rule and norm in society was not maintained strictly. Quarantine and preventing exposure to infectious diseases have been used as one of the oldest and most effective methods to control the spread of infectious diseases [15, 41, 52]. Taghrir (A) et al., 2020, showed that collective quarantine is effective in preventing the spread of COVID-19 [53]. The lack of a supportive environment was another finding of the research. Poor support and sometimes barriers in the workplace, such as not providing health supplies by the employer, not providing the necessary hygiene items in the workplace, crowded work environment, not observing health norms by the customer, client, and other colleagues were could lead to a potential disaster. It is natural that when the environment does not want the person to observe health rules, the person will not have much desire to confront the pressure and the atmosphere around him. Regarding the specific conditions of COVID-19, the performance of officials and the media has been an influential factor in all countries and has been continuously evaluated [54, 55]. The results of the study showed that the authorities, by not taking COVID-19 seriously and sometimes making fun of its consequences, broadcasting contradictory messages, different statistics and figures, setting the ground for reducing trust, made the respondents underestimate the performance of the officials and the national media. Chen et al., 2020, have shown that in times of public crises, governments must quickly and efficiently communicate crises information effectively to the members of the community. Because failure to do so will inevitably lead citizens to fear, lack of confidence and trust, non-observance of instructions and guidelines, and anxiety [54]. Abramowitz et al., 2015, also showed that the Libyan government’s various health messengers and media reports about Ebola had negative consequences for society [56]. However, in the field of information and the use of social networks and media, the speed of action of governments and responsible organizations for accurate, unambiguous, uniform, and regular information should be made compulsory to lead people to follow the health norms. In Iran, social capital is at a weak (low) level and the relationship and interaction of the government with the nation is not a two-way relationship based on cooperation, trust, and agreement. Moreover, the officials and rulers are far from the people and do not fulfill their roles and responsibilities properly. This situation is exacerbated by poor support, adaptation, and social adjustment. The people’s trust in the government is very low, and the weakness of the government in gaining the trust of the people and not organizing their participation in solving social problems creates grounds for distrust and people’s unwillingness to accept the recommendations and policies of government officials. In other words, trust at the society level is low and this trust in the government is minimal, as well as people do not follow the protocols designed and communicated by the government due to stubbornness with the government. In other words, the government lacked adequate social capital from the beginning and, by adopting inappropriate policies to combat the COVID-19 crisis, reduced this capital even more than before. At the societal level, if social cohesion is high, the level of compliance with health protocols will be better. Because people see themselves the same as and in line with the government and the society. There was not much cohesion during the outbreak of COVID-19, and people did not fulfill their responsibilities appropriately due to a lack of sense of belongingness to the society and the government. People had little cooperation with the health and medical staff. The government should work on strengthening social capital, which is based on shaping the internal cohesion of the government so that it can gain the connection and trust of people, and by strengthening communication, create empathy and strengthen the social capital and social trust of the people.

According to the results of this study and in line with other studies [57, 58], economic costs of living and lack of economic support from the government were among the economic reasons for not observing health advice. Many people were forced to continue their job due to the economic conditions of the family, unstable income, as well as the high cost of living, even in the critical situation. With the outbreak of coronavirus and falling of businesses, these pressures on families have become huge, and the support of the government and other institutions was needed at that time. However, the results of the study showed that government grants and facilities were very little and not enough to meet the basic needs of the families. These people prioritize economic need over any other issue and threat and see the inability to meet economic needs more deadly than any other disease.

The category of social and cultural factors was another major and interesting finding of the present study. Learning is the central part of every person’s life. In the present study, the results showed that people’s behavior in society affects each other and the non-observance of health norms by people can be explained regarding the observation of other people’s behavior. In Iran, since COVID-19 is not taken seriously and hence, many people do not observe the health issues related to it. Thus, this non-observance of health issues has become a model. Abramowitz et al., 2015, in examining the content of communications and transmission of Ebola-related behaviors and practices through mass media and social learning in Liberia showed that people’s behavior concerning Ebola was influenced by social learning and the behavior of other people and media [56]. Bellato’s study, 2020, discovered that people followed health and safety norms by following others, and this was significant during the COVID-19 outbreak [59].

Cultural beliefs were another reason for the lack of observance of health advice. Fatalism and non-belief in observing health norms, followed by solving problems with traditional ways, was highly prevalent in Iran. Fatalism in Iran is one of the main challenges in the field of culture for the logical perception of risk. In the present study, fatalism was one of the influential factors. These findings are consistent with the study of Taghrir (B) et al., 2020, which showed that the level of risk perception among medical students, especially interns, is low [41]. Additionally, the results of the study showed that another major reason for not observing health norms in Iranian society should be sought in the field of culture, customs, and rituals. Cultural factors have such a strong influence on the perception of risk that it practically causes the Iranian society to pay no attention to health issues. Studies in the field of infectious disease epidemiology have sought to examine the role of cultureS and social customs in diseases, such as HIV/AIDS, Ebola, SARS, and more recently COVID-19 [60–62]. In the case of COVID-19, the coincidence of its prevalence with New Year and the existence of special social customs and rituals in Iran, which have a strong normative and value aspect, making it more difficult for Iranians to observe health advice. New Year parties, visits, and close relationships with each other led to the normalization of the situation, not taking the COVID-19 seriously, and the increase of the infected cases.

### Limitations and strengths

This study is one of the first studies in Iran and most likely (based on a large search of COVID-19 articles) in Central Asia that identified the reasons for not following health tips related to COVID-19 with a qualitative method and from the perspective of high-risk business owners which can provide policymakers with useful information for proper planning to intervene to increase the observance of health issues in high-risk occupations. Variety in sampling and selection of people with different occupations was the other strength of this study.

This research also had some limitations. Finding people who met the inclusion criteria and worked, especially in high-risk occupations was a limitation of this research. Because due to quarantine and restrictions on occupational activities, some high-risk occupations were closed, and samples were more difficult to be accessed. Furthermore, some participants were reluctant to face-to-face interviews due to the outbreak. The researcher relieved their worry by observing health tips and giving gloves and masks to them. Keeping the distance with participants during the interview made some participants feel uncomfortable, and the intimate atmosphere between the researcher and the participants did not take place. Also, the interviews that took place outdoors, due to the loud noise, in some cases, reduced the quality of the recorded sound and made it difficult to transcribe. The difficulty of transcribing the interview also made the data analysis time-consuming, and in some cases, it was necessary to listen to a text several times and several people to confirm the correctness of the text, and even in several cases, the interviewees were called to confirm the transcript. It was separate from the process used to validate the data. The shortage of masks and gloves was another limitation of this research because the researcher had to spend many hours finding gloves and masks.

## Conclusion

The results of the study showed that individual, structural, economic, and socio-cultural factors contribute to the non-observance of health advice related to COVID-19 among high-risk job owners. Therefore, prevention of the spread of this disease requires intervention at various levels. In order to strengthen people’s attitudes and behavior to follow health recommendations, it is necessary to act coherently so that all different aspects of this phenomenon are considered. Individual and collective education and strengthening the health literacy of the people is necessary to make them follow the health recommendations. Awareness can be raised through various media such as television and cyberspace. Moreover, the government should take action to strengthen social capital, with the people and for the people, not acts as the only custodian of health and decides for them without sufficient knowledge of the health, economic and social needs of the people. Because when people are involved in government decisions, it is easier for them to follow the rules and regulations. This requires the government to connect and be in line with people. Hence, the government can gain the trust of the people by involving them in problem-solving and planning. It is also possible to persuade people to stay home or follow health advice when they leave home by meeting some of their economic needs and making available the hygiene products they need.

## Supplementary Information

**Additional file 1.** Checklist for questions of the interview guide.

## Data Availability

The datasets used and/or analyzed during the current study are available from the corresponding author on reasonable request.
