# Supplementary material for: Explaining the reasons for not maintaining the health guidelines to prevent COVID-19 in high-risk jobs: a qualitative study in Iran
Source: BMC Public Health. 2021 May 3;21:848. doi: 10.1186/s12889-021-10889-4 (PMC8090924; doi:10.1186/s12889-021-10889-4)
Supplement: Supplementary file 1 — Additional file 1. Checklist for questions of the interview guide. [file 12889_2021_10889_MOESM1_ESM.docx]

**Checklist for questions of the interview guide**

| No. | Questions |
| --- | --- |
| 1 | What do you know about COVID-19? Explain. |
| 2 | Explain the symptoms, ways of transmission and how dangerous COVID-19 is. |
| 3 | How do you think COVID-19 can be prevented? |
| 4 | Did you know that your job is one of the most risky jobs related to COVID-19? If yes, then why are you still working and have not stopped working? |
| 5 | Why do not you observe health issues? |
| 6 | Do you have enough access to hygienic products or are you reluctant to use them yourself? Explain. |
| 7 | Does your employer warn you about observing health issues? Does it provide you with hygiene items? Explain. |
| 8 | Do your colleagues observe health issues? Explain. |
| 9 | What do you think about the performance of officials and television about the information and warnings they provide for people? Do they encourage you to consider health issues? Explain. |
| 10 | What are the most important barriers for you to observe health issues? Explain. |
